# Applications and advances of combined fMRI-fNIRs techniques in brain functional research

**DOI:** 10.3389/fneur.2025.1542075

**Published:** 2025-03-18

**Authors:** Lirui Yang, Zehua Wang

**Affiliations:** ^1^Key Laboratory of Biomechanics and Mechanobiology, Beihang University, Ministry of Education, Beijing, China; ^2^Key Laboratory of Innovation and Transformation of Advanced Medical Devices, Ministry of Industry and Information Technology, Beijing, China; ^3^National Medical Innovation Platform for Industry-Education Integration in Advanced Medical Devices, Interdiscipline of Medicine and Engineering, Beijing, China; ^4^School of Biological Science and Medical Engineering, Beihang University, Beijing, China; ^5^Center for Medical Device Evaluation, NMPA, Beijing, China

**Keywords:** fMRI, fNIRs, multimodal neuroimaging, brain function, cognitive neuroscience, clinical applications

## Abstract

Understanding the intricate functions of the human brain requires multimodal approaches that integrate complementary neuroimaging techniques. This review systematically examines the integration of functional magnetic resonance imaging (fMRI) and functional near-infrared spectroscopy (fNIRs) in brain functional research, addressing their synergistic potential, methodological advancements, clinical and neuroscientific applications, and persistent challenges. We conducted a comprehensive literature review of 63 studies (from PubMed and Web of Science up to September 2024) using keyword combinations such as fMRI, fNIRs, and multimodal imaging. Our analysis reveals three key findings: (1) Methodological Synergy: Combining fMRI’s high spatial resolution with fNIRs’s superior temporal resolution and portability enables robust spatiotemporal mapping of neural activity, validated across motor, cognitive, and clinical tasks. Additionally, this study examines experimental paradigms and data processing techniques essential for effective multimodal neuroimaging. (2) Applications: The review categorizes integration methodologies into synchronous and asynchronous detection modes, highlighting their respective applications in spatial localization, validation of efficacy, and mechanism discovery. Synchronous and asynchronous integration modes have advanced research in neurological disorders (e.g., stroke, Alzheimer’s), social cognition, and neuroplasticity, while novel hyperscanning paradigms extend applications to naturalistic, interactive settings. (3) Challenges: Hardware incompatibilities (e.g., electromagnetic interference in MRI environments), experimental limitations (e.g., restricted motion paradigms), and data fusion complexities hinder widespread adoption. The future direction emphasizes hardware innovation (such as fNIR probe compatible with MRI), standardized protocol and data integration driven by machine learning, etc. to solve the depth limitation of fNIR and infer subcortical activities. This synthesis underscores the transformative potential of fMRI-fNIRs integration in bridging spatial and temporal gaps in neuroimaging, while enhancing diagnostic and therapeutic strategies and paving the way for future innovations in brain research.

## 1 Introduction

Understanding the complex functions of the human brain stands as one of the most captivating and challenging endeavors in contemporary science. The brain orchestrates a myriad of cognitive, emotional, and motor processes, making the elucidation of its complex mechanisms crucial for advancing both fundamental neuroscience and clinical applications. Neurological and psychiatric disorders—such as Alzheimer’s disease, depression, and autism spectrum disorder—impose significant burdens on individuals and healthcare systems worldwide, underscoring the urgent need for advanced diagnostic and therapeutic tools (1). Consequently, the scientific community has intensified its efforts to decode brain activity, spurring the development and refinement of various neuroimaging techniques.

Currently, numerous brain studies rely on non-invasive imaging modalities that each offer unique insights into brain structure and function. Techniques such as electroencephalography (EEG), positron emission tomography (PET), fMRI and fNIRs have been instrumental in mapping brain activity, understanding cognitive processes, and evaluating the efficacy of interventions for brain disorders (2). Among these, fMRI and fNIRs stand out as particularly impactful tools in cognitive neuroscience and clinical research due to their distinct advantages and complementary capabilities (3).

### 1.1 The fundamental basis of fMRI

Since its inception in the early 1990s, fMRI has been a cornerstone of neuroimaging, owing to its ability to visualize deep brain structures and its widespread adoption in both research and clinical settings.

However, fMRI is not without limitations. The technique relies on expensive, immobile equipment, and its sensitivity to motion artifacts often impedes studies in dynamic or naturalistic environments (4).

Since its inception in the early 1990s, fMRI has been a cornerstone in neuroimaging, providing high-resolution spatial maps of brain activity by detecting the Blood Oxygen Level Dependent (BOLD) signals. This technique enables researchers to localize brain regions involved in specific cognitive and sensory tasks with millimeter-level precision, covering both cortical and subcortical structures, including the hippocampus, amygdala, and thalamus. The ability of fMRI to visualize deep brain structures and its non-invasive nature have made it indispensable in cognitive neuroscience, facilitating studies on sensory processing, motor control, emotional regulation, and complex cognitive functions such as memory, attention, and decision-making (5). Furthermore, fMRI’s whole-brain coverage supports the simultaneous examination of multiple brain areas network connections, making it particularly advantageous for investigating the neural mechanisms underlying psychiatric and neurological disorders (6) and assessing brain function in longitudinal studies (7).

However, fMRI is not without limitations. The temporal resolution of fMRI is constrained by the hemodynamic response, which typically lags behind neural activity by 4–6 s (8), with a BOLD signal sampling rate generally ranging from 0.33 to 2 Hz (9). Additionally, the requirement for participants to remain motionless within the scanner environment poses challenges for studying naturalistic behaviors and limits its applicability in populations prone to movement, such as children or individuals with motor impairments. The high cost and limited accessibility of fMRI facilities also restrict its widespread use, particularly in dynamic or naturalistic environment studies.

### 1.2 The fundamental basis of fNIRs

In contrast to fMRI, fNIRs has emerged as a promising alternative capable of addressing some inherent limitations of fMRI (10). By utilizing near-infrared light (650–950 nm) to measure changes in oxygenated hemoglobin (HbO) and deoxygenated hemoglobin (HbR) concentrations on the cortical surface, fNIRs provides an indirect measure of neural activity with superior temporal resolution, often achieving millisecond-level precision (11). This flexibility allows fNIRs to capture rapid neural dynamics and makes it particularly suitable for studies involving active behaviors and naturalistic settings, such as rehabilitation exercise, social interactions, and real-world cognitive tasks (12). Furthermore, the portability and cost-effectiveness of fNIRs systems facilitate brain imaging in various settings beyond the traditional laboratory, including bedside monitoring and field studies, and expand accessibility for a wide range of populations, including infants and individuals with motor disabilities (13).

However, fNIRs also has limitations. Its spatial resolution is typically lower than that of fMRI, generally ranging from 1 to 3 centimeters, which restricts the ability to precisely localize brain activity. Moreover, fNIRs is confined to monitoring superficial cortical regions due to the limited penetration depth of near-infrared light, making it unsuitable for investigating subcortical structures (14). Extracerebral factors, such as scalp blood flow and hair, can also confound fNIRs measurements, potentially impacting data accuracy. Despite these constraints, the unique advantages of fNIRs—particularly its resilience to motion artifacts and applicability in naturalistic environments—make it an invaluable tool in neuroimaging, especially when combined with other modalities like fMRI to achieve a more comprehensive understanding of brain function.

### 1.3 Motivation of the present review

As the complexity of brain research increases, it has become evident that no single imaging modality can comprehensively capture the multifaceted nature of brain function. Multimodal approaches, which integrate different neuroimaging technologies, offer a more holistic understanding by leveraging the strengths of each technique while mitigating their individual limitations. Among these, the combined use of fMRI and fNIRs has garnered significant attention. This multimodal strategy capitalizes on fMRI’s unparalleled spatial resolution and ability to probe deep brain structures, alongside fNIRs’s temporal precision and operational flexibility (15). The integration of these modalities facilitates the simultaneous acquisition of high-resolution spatial data and real-time temporal information, providing a richer and more nuanced picture of neural activity.

**Figure 1:**
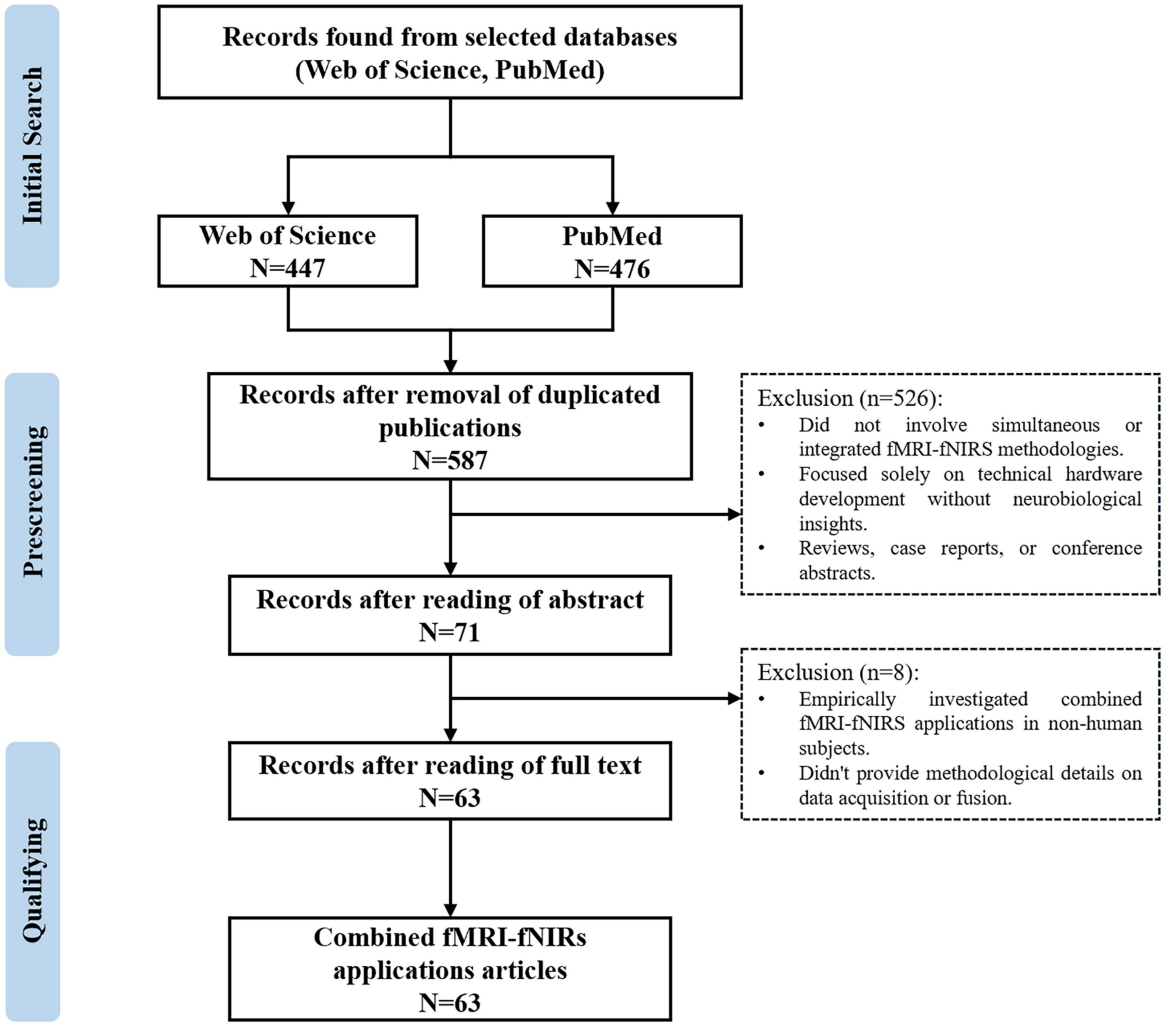
PRISMA flow diagram for the literature review and article selection.

The synergy between fMRI and fNIRs extends beyond mere data acquisition; it encompasses methodological advancements in data fusion, analysis, and interpretation. By aligning the spatially detailed fMRI maps with the temporally dynamic fNIRs signals, researchers can achieve a more comprehensive characterization of brain processes, enhancing the accuracy of neural correlates and connectivity analyses. Furthermore, this combined approach is particularly advantageous in clinical settings, where the portability of fNIRs allows for bedside monitoring of patients alongside the detailed structural and functional insights provided by fMRI (16). In this study, the search for relevant peer-reviewed articles describing the combined use of fNIRs and fMRI design was conducted on PubMed and Web of Science as literature sources. The following keyword combinations were used in the literature search: ((fNIRs) OR (NIRs) OR (functional near-infrared spectroscopy) OR (near-infrared spectroscopy)), AND ((fMRI) OR (MRI) OR (Functional Magnetic Resonance Imaging) OR (Magnetic Resonance Imaging)), AND ((integration) OR (combination) OR (multimodal imaging)). Only articles that were published in English through September 30, 2024, were included, identifying approximately 600 articles (Figure 1). After careful screening of abstracts and full texts, 63 studies were selected that focused on the simultaneous or integrated use of fMRI and fNIRs in neuroimaging research. These studies were meticulously analyzed, yielding critical insights into integration techniques, experimental paradigms, and data analysis methods. The review spanned diverse applications, ranging from cognitive and motor tasks to social interactions and clinical diagnostics. Various modes of integration were observed, including synchronous data acquisition and asynchronous data acquisition. This comprehensive review highlights not only the current state of combined fMRI-fNIRs research but also identifies emerging trends and future research directions, providing a detailed overview of both advancements and challenges in this evolving field.

## 2 The advantages of the integrating of fMRI and fNIRs

Integrating multiple neuroimaging modalities has become increasingly prevalent in brain research, driven by the recognition that no single technique can fully capture the complexity of neural activity. Combining modalities, such as EEG with fNIRs, or PET with fMRI, has demonstrated significant advantages in expanding the depth and breadth of brain function analysis. Among these, the simultaneous use of fMRI and fNIRs has garnered considerable attention due to their complementary strengths and potential for synergistic insights (17).

### 2.1 Significance of integrating fMRI and fNIRs

Both fMRI and fNIRs measure hemodynamic responses related to neural activity, however, they fundamentally differ in their spatial resolution, temporal resolution, resistance to motion interference, and portability. The integration of fMRI and fNIRs raises important questions regarding the redundancy and complementary value of combining similar hemodynamic-based techniques. Since both fMRI and fNIRs reflect blood oxygenation changes, with fMRI offering high spatial resolution across the entire brain, their combined use has become essential for validating the efficacy and reliability of fNIRs technology. Throughout the development of fNIRs, synchronized multimodal imaging has served as a crucial approach for confirming the utility of near-infrared imaging techniques in human brain science research (18).

fMRI provides high spatial resolution, enabling detailed localization of brain activity throughout the brain, including deep structures. In contrast, fNIRs, often referred to as a “wearable fMRI,” provides high temporal resolution, is resistant to motion artifacts, and is portable, allowing for the real-time monitoring of rapid cortical hemodynamic changes in more naturalistic settings. fNIRs’s ability to capture rapid hemodynamic fluctuations complements fMRI’s precise spatial mapping, enabling researchers to correlate real-time cortical activity with detailed brain region localization (19). This complementary nature suggests that integrating fMRI and fNIRs could leverage the strengths of both modalities, providing a more comprehensive picture of neural dynamics and facilitating studies that require movement or interaction, which are challenging within the confines of an MRI scanner.

The integration of fMRI and fNIRs has emerged as a powerful approach to advance our understanding of brain function in both clinical and neuroscience research. Applying the fNIRs hyperscanning approach to the synchronous integration of fMRI and fNIRs not only deepens the neuroscientific understanding of interpersonal interactions but also provides new tools and methods for cross-brain research in social behavior. This multimodal approach not only bridges the gaps left by each individual technique but also opens new avenues for understanding the intricate dynamics of neural activity in both controlled and naturalistic environments (20).

### 2.2 Integrating fMRI and fNIRs in clinical neurological diseases

In clinical settings, the combined use of fMRI and fNIRs enhances the diagnosis and management of neurological and psychiatric disorders. This integration is particularly valuable for conditions where real-time monitoring of brain function during naturalistic behaviors is crucial.

Stroke and traumatic brain injury (TBI) involve complex neural impairments. fMRI is essential in mapping affected brain areas and assessing damage extent in these conditions, yet its temporal limitations and sensitivity to motion artifacts reduce its effectiveness in dynamic, real-world settings (21, 22). fNIRs complements fMRI by allowing continuous, real-time monitoring of cortical activation, particularly useful in rehabilitation tasks and settings requiring movement or social interaction (23, 24). This combined imaging approach provides insights for personalized interventions in stroke and TBI and informs targeted therapies.

For disorders like Alzheimer’s, Parkinson’s, attention-deficit hyperactivity disorder (ADHD) and psychiatric conditions such as mood disorders and schizophrenia, fMRI reveals changes in neural connectivity (25–27), while fNIRs enhances functional assessment in outpatient settings (28–30). The integration facilitates early detection, tracks disease progression, and refines personalized treatment strategies, especially during real-world therapeutic interventions.

Additionally, chronic pain and neurodevelopmental studies in young children benefit from fMRI-fNIRs integration. fMRI identifies brain regions involved in pain perception (31), while fNIRs enables real-time monitoring during pain stimuli and is a flexible alternative for studying young children’s natural behaviors (32, 33). Together, these techniques improve understanding of pain mechanisms, brain development, and early intervention opportunities.

### 2.3 Integrating fMRI and fNIRs in neuroscience

The combined use of fMRI and fNIRs has greatly advanced neuroscience research, deepening our understanding of complex brain functions such as social cognition, neuroplasticity, and brain connectivity. This integration allows for the study of neural processes in both controlled and naturalistic settings, where fMRI provides high spatial resolution and fNIRs offers portability and real-time monitoring.

Specifically, the synergistic use of fMRI and fNIRs is particularly valuable in cognitive neuroscience (34), with fMRI identifying neural networks and fNIRs capturing the temporal dynamics of attention, memory, and decision-making. In studies of neuroplasticity, fMRI detects long-term structural changes (35), while fNIRs monitors short-term hemodynamic responses during learning and rehabilitation (36).

Furthermore, this multimodal integration has been especially influential in research on emotional processing (37), connectivity patterns (38), and real-world behaviors (39), offering insights that traditional lab settings may miss. Furthermore, the integration of fMRI and fNIRs drives methodological innovations, advancing data fusion and analysis to yield more reliable neuroimaging findings (40). Overall, this multimodal approach deepens our understanding of cognition, emotion, and social interaction, advancing both the theoretical and practical applications of neuroscience.

## 3 Hybrid method

### 3.1 The combined application modes of fMRI and fNIRs

The combined use patterns of fMRI and fNIRs can be broadly categorized into synchronous and asynchronous detection modes, each catering to distinct research methodologies and objectives. Table 1 systematically categorizes various studies that have employed combined fMRI-fNIRs methodologies, elucidating the diverse patterns of utilization and procedural implementations. Table 2 illustrates the advantages and limitations of synchronous and asynchronous detection. By consolidating these patterns, these tables facilitate a comprehensive understanding of how fMRI and fNIRs can be synergistically leveraged to overcome the limitations inherent to each modality when used independently.

**Table 1:** Overview of combined use patterns in fMRI and fNIRs studies.

Detection mode	Research method/problem	Studies
Synchronous detection	Spatial localization	fMRI assists fNIRs in providing precise localization of cortical regions of interest, enhancing the overall spatiotemporal accuracy of cortical neural mapping (41, 43).
	Validation of efficacy	Concurrent fMRI and fNIRs validated fNIRs’s accuracy in monitoring cortical hemodynamics, enhancing spatiotemporal interpretation of brain activity (41, 42, 44–49, 85–93).
	Mechanism discovery	Synchronous fMRI and fNIRs enabled precise, real-time analysis of cortical dynamics, enhancing understanding of NVC and brain connectivity (50–54, 59, 67, 68, 71, 90).
Asynchronous detection	Validation of efficacy	These studies used separate fMRI and fNIRs to confirm fNIRs’s reliability in tracking cortical activity, showing spatial–temporal congruence across tasks (56–58, 62, 64, 69, 70, 73, 74, 94–101).
	Mechanism discovery	These studies used asynchronous fMRI and fNIRs to explore motor, cognitive, and social neural mechanisms, enhancing insights into brain connectivity and NVC (55, 58, 60, 61, 91, 92, 101–106).

**Table 2:** Advantages and limitations of synchronous and asynchronous detection.

Detection mode	Advantages	Limitations
Synchronous detection	Offers real-time integration, ensuring that brain activity is completely consistent. Facilitates spatial localization, aiding in the precise identification of active brain regions. Provides comprehensive brain function information by accurately mapping neural activity and temporal dynamics. Enables validation of findings through comparison of results from both technologies, easier to verify validity.	The experimental paradigms and scenarios that can be adopted are limited. There are compatibility requirements between the two technologies (fNIRs must adapt to magnetic shielding environments and potential safety risks and interference need to be controlled). Subjects must accommodate fNIRs emitter and detector sensors within the confined space of the MRI head coil, which may affect comfort and objectivity. Overcoming attenuation of near-infrared light in long fiber setups and controlling data artifacts is necessary to ensure reliable brain function imaging.
Asynchronous detection	It allows multiple measurements at different time points and scenarios, enhancing the flexibility of experimental design. It supports research on mechanism discovery, avoiding potential interference between the two technologies and not being limited by simultaneous data acquisition. Offers a wide range of experimental paradigms, suitable for longitudinal studies such as long-term monitoring and rehabilitation assessment. Does not require the simultaneous operation of two costly devices, making it more feasible in terms of cost and technology.	Spatial localization is challenging, with high demands for data correction. Data integration is complex, potentially introducing additional errors. Subject’s state may vary at different time points, affecting data consistency. Controlling experimental conditions is difficult (such as environmental factors).

#### 3.1.1 Synchronous detection

Synchronous detection involves the simultaneous acquisition of fMRI and fNIRs data within the same experimental session. This approach allows for real-time correlation and integration of the spatially precise fMRI signals with the temporally sensitive fNIRs measurements, providing a holistic view of neural dynamics. The brain’s activities are complex and change all the time. Even though the brain’s activities are performing the same task at any time, there will always be deviations. If collected synchronously, data on brain activity in the same state can be obtained. Synchronous detection is primarily utilized to address research areas related to spatial localization, validation of efficacy, and mechanism discovery.

The synchronous integration of fMRI and fNIRs significantly enhances the spatial accuracy of hemodynamic measurements by combining fMRI’s millimeter-level spatial resolution with fNIRs’s capacity to capture rapid cortical hemodynamic changes (41, 42). Hocke et al. (43) optimized a multimodal fMRI and fNIRs probe, achieving ultrahigh-resolution mapping by leveraging the complementary strengths of both modalities. This innovation enables precise spatial and temporal mapping, highlighting the efficacy of integrated fMRI/fNIRs systems for enhanced brain mapping, particularly through improved localization and sensitivity profiles. Collectively, these advancements emphasize the potential of synchronous detection to refine spatial precision in fNIRs data, making it a valuable tool for detailed brain mapping.

Whether it is the early emergence of fNIRs or the current development of new fNIRs technologies, they are all being verified in conjunction with MRI, which is a very important and indispensable link. A critical application of synchronous detection lies in validating the efficacy of fNIRs by directly comparing its signals with those obtained from fMRI (44–46). Studies confirm fNIRs’s reliability alongside fMRI, demonstrating spatial–temporal concordance in motor, cognitive, and clinical tasks, highlighting its robustness in neuroimaging (47–49).

Synchronous detection is instrumental in mechanism discovery, particularly in elucidating the relationship between brain activity and blood flow, known as neurovascular coupling (NVC), and the hemodynamic response function (HRF), which describes the changes in blood flow following neural activity. Studies by Okamoto et al. (50) and Schroeter et al. (51) highlight fNIRs’s effectiveness in naturalistic tasks and its ability to capture hemoglobin dynamics in conjunction with fMRI, enhancing understanding of NVC. Further research by Heinzel et al. (52), Muthuraman et al. (53), and Liu et al. (54) underscores fNIRs’s ability to capture non-neuronal components of the BOLD signal, assess effective connectivity, which refers to the influence of one brain region over another, and even monitor activity in deeper brain areas. Additionally, studies by Vijayakrishnan Nair et al. (55) demonstrate its applicability in cerebrovascular assessments and personalized neuroimaging. Collectively, these studies validate fNIRs’s potential alongside fMRI for detailed neurovascular insights.

#### 3.1.2 Asynchronous detection

Asynchronous detection refers to the sequential or separate acquisition of fMRI and fNIRs data within the same study or across different sessions. This approach offers distinct advantages and limitations that are crucial for its application in neuroimaging research. Asynchronous detection allows researchers to tailor each imaging session to the specific requirements of fMRI and fNIRs, offering better subject comfort without the constraints of simultaneous data acquisition. Conducting fMRI and fNIRs sessions separately can simplify the experimental setup, reducing technical and cost challenges associated with simultaneous measurements and avoiding potential interference between modalities. But the primary limitation of asynchronous detection is the inability to capture neural activity simultaneously with both modalities. This temporal separation can introduce variability due to changes in the subject’s physiological or psychological state between sessions, potentially confounding data interpretation. Despite its limitations, asynchronous detection plays a vital role in validating the efficacy of fNIRs across diverse experimental contexts and populations. This mode is particularly advantageous for validating the efficacy of fNIRs in diverse experimental contexts and populations and for exploring mechanisms that do not necessitate real-time data integration. Asynchronous detection is primarily utilized for validation of efficacy and mechanism discovery.

Asynchronous detection is widely employed to establish the reliability and applicability of fNIRs in motor, cognitive, and clinical tasks, and brain-computer interface studies (56–58). By demonstrating high spatial–temporal concordance with fMRI, asynchronous detection supports fNIRs as a valuable tool for continuous brain monitoring and longitudinal studies (59–61). Additionally, this mode allows for separate yet complementary analyses, facilitating insights into neurovascular mechanisms and the physiological basis of hemodynamic responses (62–64).

#### 3.1.3 Detection modes analyses

**Figure 2:**
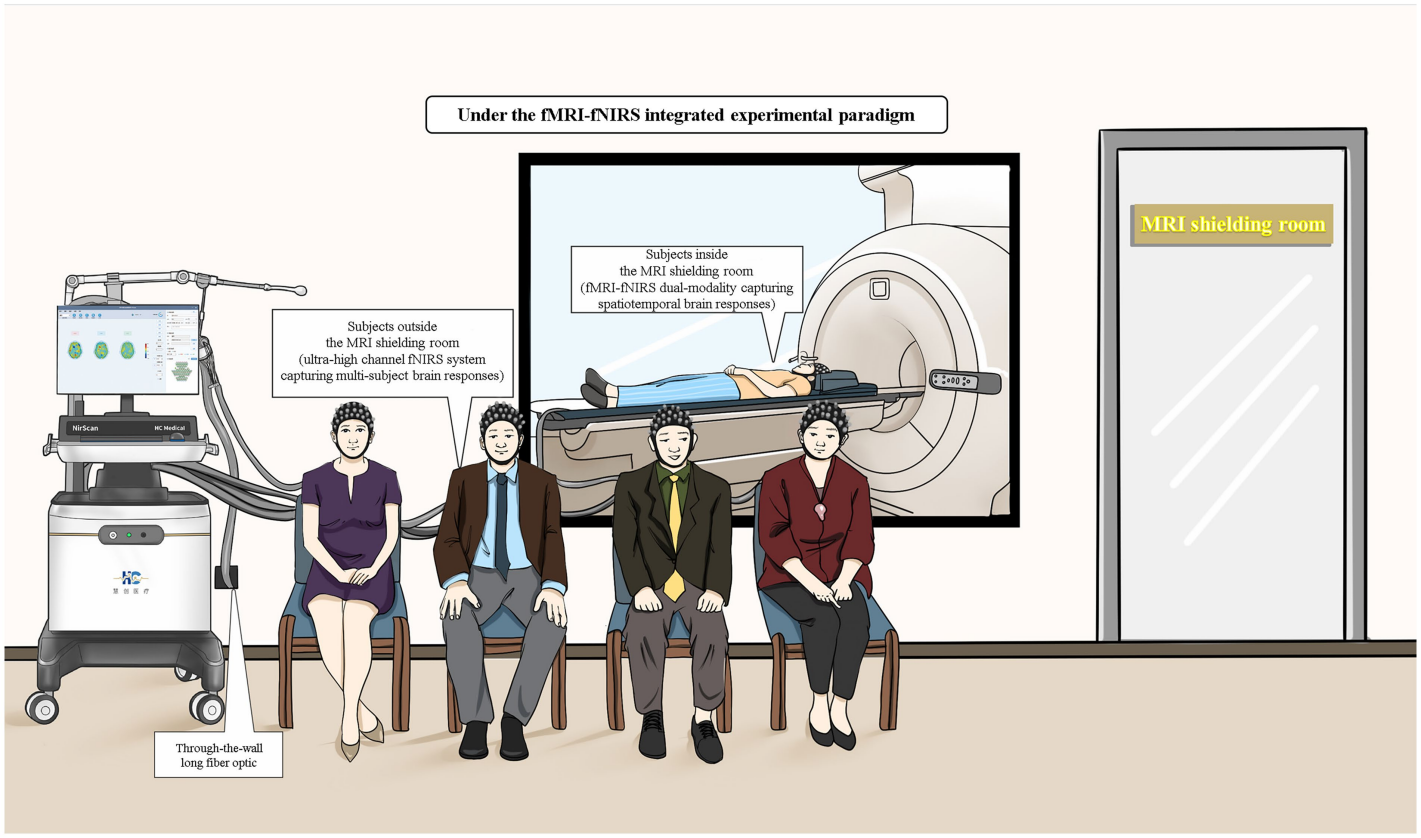
Schematic diagram of fMRI-fNIRs combined experimental tasks (including fMRI-fNIRs dual-modality acquisition of brain spatiotemporal response in a shielded room and ultra-high channel fNIRs acquisition of multi-person brain response outside a shielded room).

The combined use of fMRI and fNIRs, through synchronous and asynchronous detection modes, offers a robust and flexible framework for advancing neuroimaging research. Synchronous detection excels in real-time integration and spatial localization, making it invaluable for studies requiring precise mapping of neural activity alongside temporal dynamics. Asynchronous detection provides the necessary flexibility for validating fNIRs across diverse experimental conditions and populations, facilitating mechanism discovery without the constraints of simultaneous data acquisition. fNIRs devices are portable and have strong resistance to motion interference. When used synchronously, their advantages cannot be fully utilized. New detection methods such as partially synchronized superscanning technology only one person wears the fNIRs device in the MRI room, while the others wear the fNIRs device outside (Figure 2). In this new methods, both synchronous and asynchronous combinations are used, the advantages of both can be fully utilized. These integrated use patterns enhance the robustness, reliability, and applicability of neuroimaging studies, paving the way for more comprehensive investigations into brain function and dysfunction (65–67).

### 3.2 Experimental design

In studies combining fMRI and fNIRs, a well-designed experiment is essential to maximize the complementary advantages of these imaging modalities, capturing brain activation characteristics and network features to enable precise spatial localization, efficacy validation, and exploration of brain mechanisms. Although resting-state paradigms are commonly used, appropriate task-based paradigms may also be employed. The choice of experimental design depends on the mode of fMRI-fNIRs integration. In asynchronous modes, the design follows considerations for each imaging modality independently, capitalizing on fNIRs’s broader applicability. For synchronous modes, however, task paradigms compatible with MRI are preferred. Following a review of over 50 studies on combined fMRI-fNIRs applications, Table 3 presents a comprehensive summary of the experimental paradigms and study designs used in these works. The table categorizes the studies by task type, including resting-state, sensory stimulation, motor, cognitive, and language tasks, as well as specific design within some paradigms. The table provides a foundational reference for designing multimodal studies.

**Table 3:** Task in fMRI-fNIRs research.

Task		Asynchronous detection		Synchronous detection
		fNIRs	fMRI	fNIRs-fMRI
Resting-state tasks		(41, 67, 75, 93)	(41, 67, 75, 93)	(46, 55, 68, 71, 85, 93)
Motor tasks	Finger or toe motor task	(50, 58–60, 69, 70, 73, 94, 96, 99)	(50, 57–60, 69, 70, 73, 94, 96, 99)	(41, 44, 46–50, 53, 73, 86, 107)
	Body motor task (including limb movement)	(57, 91, 105)	(91, 105) (only slight wrist flexion and extension from a neutral position).	None
	Motor imagination task	(69, 91, 95, 98, 99)	(69, 91, 95, 98, 99)	√
Cognitive tasks	N-back task	(61)	(61, 97)	(49)
	VFT task	(59)	None	(43, 49)
	Stroop task	(97)	(97)	None
	Go/No-Go task	√	√	(54)
	Others			(42, 43, 52, 54)
Visual tasks	Visual stimulation task	(73, 106)	(73, 106)	(45, 51, 93, 108)
	Eye movement task	(56)	(56)	√
Auditory tasks	Verbal stimulation task	(104, 106)	(104, 106)	(89)
	Verbal imagination task	√	√	(92)
Breath hold tasks		(102)	(102)	(62, 87, 90)
External stimulus tasks		(101, 106)	(106)	(88)
Dual tasks		(63)	(63)	None

The choice of experimental paradigms plays a crucial role in multimodal integration studies, as it significantly influences the ability to maximize the advantages of this integration. Selecting an appropriate paradigm depends largely on the research objectives, such as whether the study involves synchronous or asynchronous integration. For synchronous integration, researchers must decide on the specific experimental mode to be employed, while for asynchronous integration, the emphasis is on optimizing its advantages by adopting paradigms suited to this approach. In the early stages of research, when the effectiveness of the experimental approach is uncertain, validation studies often utilize synchronous integration. Synchronous paradigms require compatibility across modalities, with a primary focus on those suitable for fMRI. Commonly adopted paradigms include resting-state conditions and simple motor tasks, such as finger-tapping exercises, which are frequently chosen for their compatibility and ease of implementation. For studies focusing on spatial localization, synchronous detection can effectively address localization tasks, while asynchronous integration allows for broader applications. Motion-based experimental paradigms are frequently utilized in asynchronous integration. During synchronous detection, spatial localization tasks are executed using paradigms involving small-scale movements. Following localization, asynchronous integration leverages the flexibility of fNIRs to explore more extensive and complex whole-body motion-related brain activities. Here, the choice of paradigms involves fine-tuned small-scale movements for synchronous integration, enabling fNIRs to monitor larger-scale activities effectively in asynchronous conditions. When investigating underlying mechanisms, more diverse experimental paradigms come into play. Some paradigms, however, are not fully compatible with fMRI. In such cases, traditional paradigms, such as the n-back task or the Stroop test, can be adapted to accommodate fMRI’s requirements. For instance, responses that traditionally involve vocal answers can be modified to use button-pressing methods, which are more suitable for fMRI detection. Similarly, motor tasks, including limb movements, must be carefully constrained for fMRI compatibility. For example, in body motor tasks (including limb movements), wrist joint tasks are typically restricted to slight flexion-extension movements from a neutral position to ensure compatibility with the scanner’s operational constraints.

### 3.3 Data processing methods

**Figure 3:**
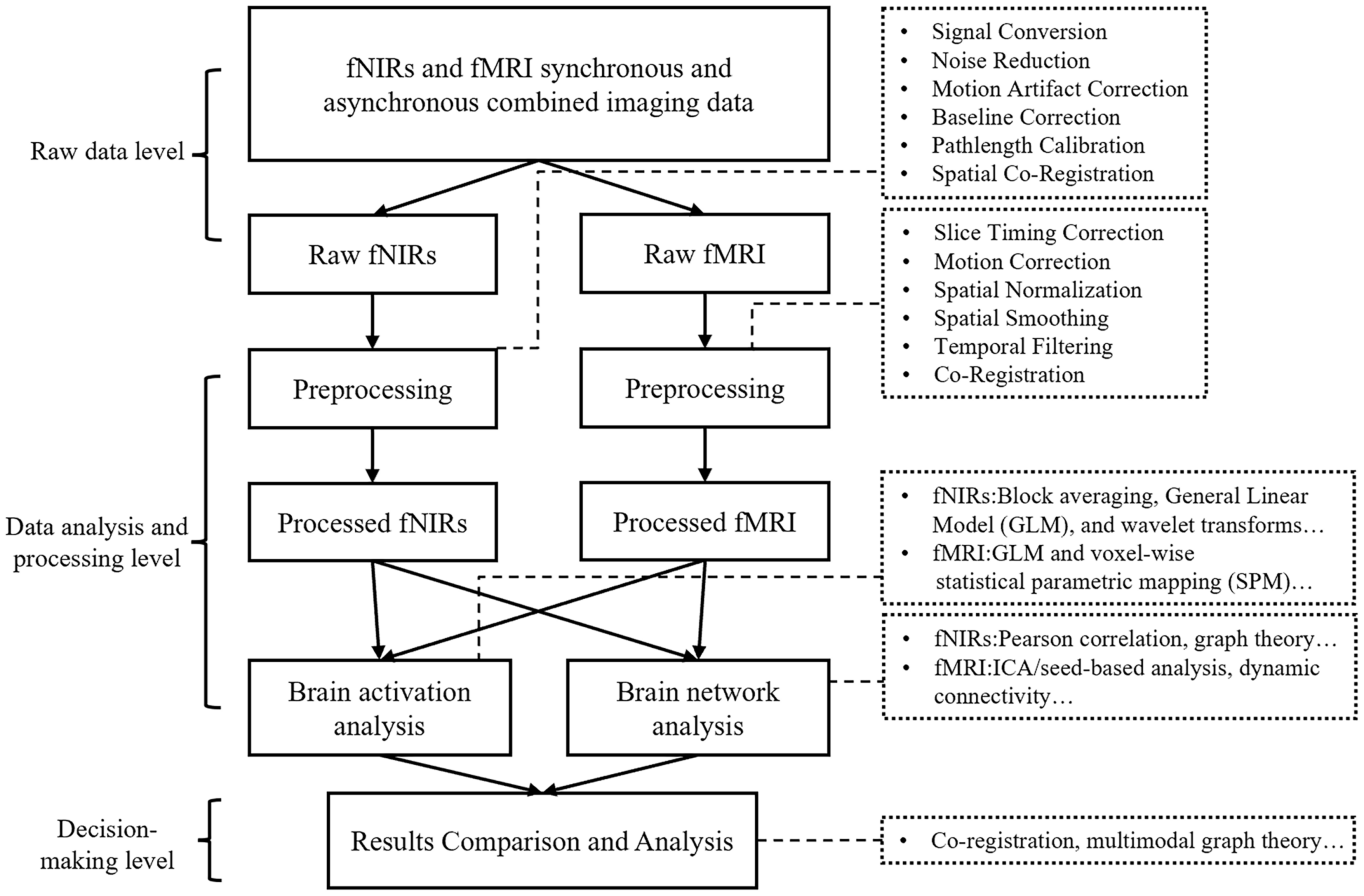
The schematic of fNIRs-fMRI data processing research methods.

The integration of fMRI and fNIRs has emerged as a robust multimodal approach in cognitive neuroscience, providing complementary insights into cerebral hemodynamics and neural activity. By combining the spatial precision of fMRI with the temporal sensitivity and practical versatility of fNIRs, researchers can investigate brain dynamics and NVC in unprecedented detail. When conducting multimodal data, there are generally two primary approaches to data processing. One is to analyze the joint imaging data separately to obtain qualitative results, and then cross-integrate the processed results at the decision-making level. The other is to cross-integrate the joint imaging data at the data level or during the processing to obtain a common result. When conducting joint imaging research on fMRI and fNIRs, it is difficult to cross-integrate the data of the two modalities at the raw data level or analysis and processing stage, given the great differences between the collected fMRI and fNIRs data. Typically, the two types of data are processed separately and subsequently cross-integrated at the decision-making level (Figure 3). Effective data processing methods are essential to harness the complementary strengths of both techniques, enabling a more comprehensive understanding of brain function. This section provides a comprehensive review of the data processing methodologies employed in combined fMRI-fNIRs studies, underscoring the critical steps necessary for effective multimodal neuroimaging analysis.

The datasets of some of the research papers are already public. For example, you can visit https://leondlotter.github.io/MAsync/MAsync_analyses.html and https://osf.io/hf4cr/ to get a comprehensive understanding (61, 67).

#### 3.3.1 Separate data processing and analysis

Processing fMRI data involves a sequence of preprocessing steps to refine the BOLD signals, which indirectly indicate neural activity through fluctuations in deoxyhemoglobin concentrations. The initial step, slice-timing correction, rectifies temporal inconsistencies arising from the sequential acquisition of brain slices, ensuring temporal alignment across the entire dataset. This is followed by motion correction, which mitigates artifacts resulting from head movements by applying rigid-body transformations to stabilize the brain images. Spatial normalization subsequently maps individual brain images onto standardized anatomical templates, such as the Montreal Neurological Institute (MNI) or Talairach atlases, thereby facilitating cross-subject and group-level analyses. To enhance the signal-to-noise ratio, spatial smoothing is performed using a Gaussian kernel, which helps in reducing anatomical variability and improving statistical power. Additionally, temporal filtering is employed to eliminate physiological noise and signal drifts outside the frequency range pertinent to neuronal activity, thereby isolating meaningful BOLD fluctuations. Since fNIRs and fMRI data are cross-integrated at the final decision layer, the activation results and network results obtained from fNIRs and fMRI are usually compared at the decision layer. For pre-processed fMRI data, block averaging, GLM and other analysis methods are usually used when analyzing brain activation, and functional connectivity, effective connectivity, default network and other analysis methods are usually studied when analyzing brain networks (68, 69).

Processing fNIRs data likewise involves a series of preprocessing steps, especially in signal noise and artifact handling. Effective denoising techniques, such as wavelet filtering and independent component analysis (ICA), are employed to remove physiological noise arising from cardiac pulsations and respiratory fluctuations. Motion artifact correction is critical, as movements can introduce significant distortions, strategies like spline interpolation and correlation-based methods are utilized to mitigate these effects. Moreover, Baseline correction is performed to eliminate slow signal drifts, either by subtracting pre-stimulus baselines or applying detrending algorithms, ensuring that the data accurately reflect neural activity. Accurate optical pathlength estimation is essential to account for individual variability in tissue absorption and scattering properties, enabling precise quantification of changes in HbO and HbR concentrations. After preprocessing, activation analysis and network analysis can be performed. The activation analysis of fNIRs is similar to that of fMRI, which usually uses analytical methods such as block averaging, GLM, and wavelet transform, etc. It can also perform the analysis of functional and effector network connections, which can be used to further go on to study the mechanisms of brain sciences based on a variety of parameters (68, 70).

#### 3.3.2 Data processing method in combined fMRI-fNIRs studies

The integration of fMRI and fNIRs data necessitates meticulous strategies to reconcile their differing data characteristics. Preprocessing remains largely modality-specific due to the distinct signal acquisition mechanisms inherent to each technique. Spatial co-registration is a pivotal step, aligning the placement of fNIRs probes with anatomical references of fMRI images, such as three-dimensional (3D) digitizers or individualized MRI-based head models, which significantly enhance spatial accuracy (71, 72). Activation analyses within the integrated framework involve generating and overlaying activation maps from both fMRI and fNIRs to assess concordance in task-related brain responses. Metrics such as correlation coefficients and overlap indices are employed to quantify the consistency of activation patterns, thereby validating findings and highlighting the complementary insights provided by each modality. Furthermore, functional connectivity analyses are significantly enhanced by this integration, as functional connectivity matrices are constructed by correlating signals from regions of interest (ROIs) identified in both fMRI and fNIRs datasets. Graph theoretical approaches are then utilized to characterize network properties, including global efficiency, local efficiency, and node centrality. These metrics facilitate comparative analyses that can identify modality-specific network features, thereby enriching our understanding of brain network organization and dynamics (69, 73).

## 4 Challenges and future directions

While the integration of fMRI and fNIRs holds significant promise for advancing our understanding of brain function, several limitations and challenges must be addressed to fully realize its potential. This section critically examines these limitations, discussing their implications for research outcomes, and proposes future directions to overcome these challenges.

### 4.1 Challenges

**Figure 4:**
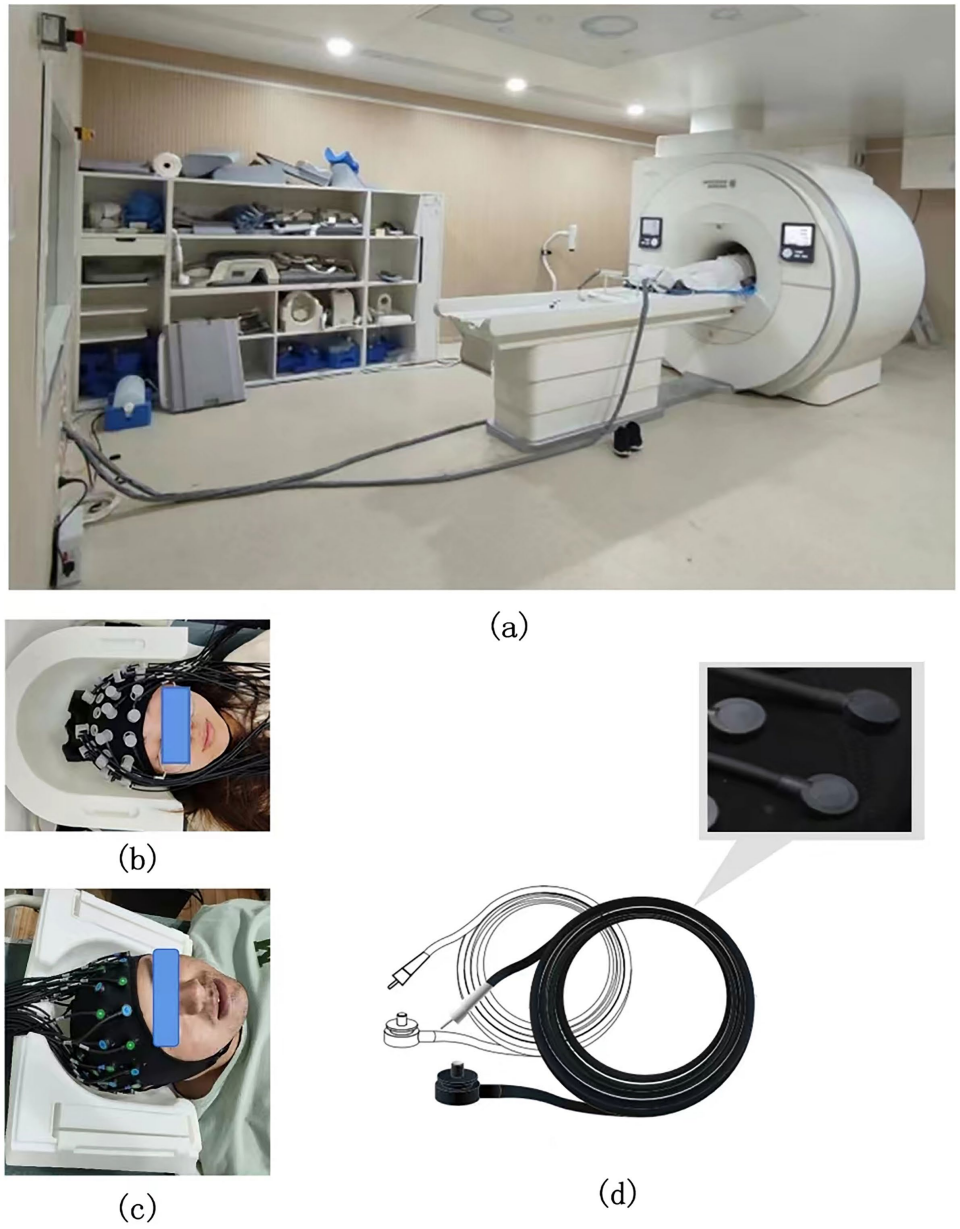
**(a)** Schematic diagram of the extra-long optical fiber in the fNIRs-fMRI simultaneous acquisition experiment. **(b,c)** Schematic diagram of the head cap and head detail reproduction in the fNIRs-fMRI simultaneous acquisition experiment. **(d)** The acquisition probe compatible with fMRI in the fNIRs-fMRI simultaneous acquisition experiment (it has the following properties: full brain coverage, ultra-thin probe, extra-long optical fiber, scalp fit, and no interference from magnetic fields).

Firstly, the synchronization of data acquisition in the integration of fMRI and fNIRs studies imposes stringent requirements on hardware compatibility and resources. fMRI operates within a strong magnetic field, necessitating meticulous control to mitigate potential electromagnetic interference. Meanwhile, fNIRs employs optical sensors, that may interact with the magnetic field of the MRI, raising safety concerns and introducing data artifacts during simultaneous acquisition. The combined use requires access to MRI facilities and specialized equipment for simultaneous data acquisition, which entails high operational costs and demands specialized skills (66). The development of fNIRs systems that are compatible with high-field MRI environments, utilizing non-metallic optodes and advanced shielding materials, remains a critical area of ongoing research (Figure 4d). Currently, the common solution is to place the fNIRs host in the main control room or the computer room, which is connected with the fNIRs cap through the ultra-long optical fiber (>8 m) to complete synchronous detection. Ultra-long optical fiber will bring signal attenuation, thus ensuring the quality of signal detection is also an urgent problem to be solved in synchronized imaging (Figure 4a).

In addition, ensuring the spatial compatibility between the MRI head coil and the fNIRs head cap remains a significant challenge. The head coil is essential for MRI head imaging, and reducing the spatial footprint of the fNIRs head cap is a critical consideration. Currently, some fNIRs manufacturers, such as Huichuang, have introduced offset probe designs to minimize the space occupied by the fNIRs head cap, enabling better adaptability to various head coils (Figures 4b,c). However, further efforts are required to reduce the spatial footprint of fNIRs probes and optical fibers, ensuring seamless integration with MRI systems.

Differences in experimental designs, task paradigms, and data processing techniques across studies present challenges for reproducibility and comparability (74). Variability in factors such as optode placement, signal processing methods, and statistical analyses can lead to inconsistent results. The absence of standardized guidelines for conducting combined fMRI-fNIRs research hinders the ability to generalize findings and draw definitive conclusions about brain function and dysfunction. Participants must remain still within the confined space of an MRI scanner, which constraint limits the types of tasks and behaviors that can be studied. Current fMRI-fNIRs integration studies predominantly rely on resting-state and simple motor tasks, limiting their applicability to more complex cognitive and social interaction scenarios. To fully leverage the advantages of combining fMRI and fNIRs, relying solely on simultaneous acquisition is insufficient. While simultaneous acquisition aligns temporal and spatial information from both modalities, it may limit fNIRs’s inherent robustness against motion artifacts.

Finally, aligning and synchronizing data from these modalities necessitates sophisticated computational methods and algorithms (11). Differences in data processing pipelines, such as signal filtering and statistical analysis, often lead to inconsistent results across studies, the lack of standardized protocols for multimodal data fusion complicates the interpretation of combined datasets. Furthermore, the limited penetration depth of near-infrared light restricts fNIRs to monitoring cortical surfaces, leaving subcortical structures inaccessible (44). Although some research try to improve the detection depth and resolution of fNIRs, the improvement is limited by its imaging principle. This limitation means that fNIRs cannot capture the full extent of neural activity, particularly in deep brain regions critical for various cognitive and emotional processes.

### 4.2 Future directions

Current fMRI-fNIRs integration studies predominantly rely on resting-state and simple motor tasks, limiting their applicability to more complex cognitive and social interaction scenarios. Integrating simultaneous acquisition with fNIRs hyperscanning, however, offers a novel methodological approach that can open new avenues for studying interactive and dynamic processes in social neuroscience. Hyperscanning addresses these challenges by recording neural activity across interacting participants, offering insights into inter-brain synchronization and its relation to cognitive and emotional processes. fNIRs hyperscanning excels in portability and tolerance to movement, making it suitable for ecological settings and a broader range of populations, including children and clinical groups. Studies have shown its efficacy in capturing brain-to-brain connectivity during cooperative and competitive tasks, emphasizing its potential to explore how social bonds and group dynamics form and evolve. Integrating these methods can improve the robustness of findings by combining fMRI’s spatial precision with fNIRs’s temporal resolution and ecological applicability (75, 76).

Furthermore, the constrained penetration depth of near-infrared light confines functional near-infrared spectroscopy (fNIRs) to assessing hemodynamic changes in cortical surfaces, while subcortical brain regions remain inaccessible due to insufficient photon penetration. Although efforts have been made to enhance detection depth and spatial resolution through methodological optimizations, these advancements remain fundamentally restricted by the inherent physical principles of optical attenuation and scattering in biological tissues. However, the combined fMRI-fNIRs approach may extend the functional imaging range of fNIRs to subcortical regions, complementing the spatial precision of fMRI and enabling a more holistic view of brain activity, combining fNIRs-fMRI with complementary modalities such as EEG may also provide a more comprehensive picture of brain activity (70).

In the end, establishing standardized guidelines for experimental design, data acquisition, and data processing in combined fMRI-fNIRs studies is essential. Collaborative efforts to create shared databases and repositories can facilitate data comparison and meta-analyses, promoting consistency and reliability in research findings. Machine learning is now a commonly used method. Currently, there are many studies on the application of machine learning in both fNIRs (77–79) and fMRI (80–82) imaging modalities. Implementing sophisticated computational algorithms and machine learning techniques can improve the fusion of fNIRs and fMRI data, enabling more accurate and meaningful interpretations of multimodal datasets. For instance, Jihyun Hur et al. have made significant strides by employing machine learning models and data augmentation to predict fMRI markers from fNIRs data (83). Lingkai Tang et al. have made significant advances by using graph convolutional networks (GCNs) to predict cortical-thalamic connectivity from fNIRs data. They analyzed datasets from healthy adults and neonates with brain injuries, using fNIRs for cortical measurements and fMRI as the connectivity ground truth. This integration allowed them to infer subcortical activity, overcoming fNIRs depth limitations and enhancing its clinical use for monitoring brain function in critically ill patients (84). In the future, there will be more and even joint use of these, we may use machine learning in several aspects. First, we apply machine learning to the two data separately in the data processing layer to make a better judgment in the decision-making layer later. Second, fMRI has data on deep nuclei, but the disadvantage of fNIRs is that it does not have data on deep nuclei. If we can put the data of these two together, we can generate fNIRs data with deep nuclei. If there is only a small amount of MRI data or standard brain maps plus fNIRs data in the future, we can speculate on some situations of deep nuclei. Designing experimental setups that enhance participant comfort, such as using more ergonomic equipment and creating realistic task environments, can reduce motion artifacts and improve data quality. Developing cost-effective fNIRs and fMRI equipment can make combined fMRI-fNIRs research more accessible, and applying the combined fMRI-fNIRs approach to a wider range of neurological and psychiatric conditions can deepen our understanding of brain function across contexts, advancing both clinical and research applications.

## 5 Conclusion

The integration of fMRI and fNIRs provides a powerful neuroimaging approach, integrating high spatial resolution with superior temporal resolution. In 2017, previous reviews mainly focused on the basic principles, advantages and limitations of fMRI and fNIRs, as well as the validation of their combination in research and the in-depth understanding of BOLD signals. They analyzed how fNIRs can complement the shortcomings of fMRI from a technical perspective and discussed future research directions (66). This review comprehensively analyses the integration of fMRI and fNIRs, emphasizing the specific progress and application of the combination of fMRI and fNIRs in recent years. It not only analyses the application and progress of the combination of fMRI and fNIRs in brain function research, covers specific application cases in multiple fields, but also discusses in detail the combination mode, experimental paradigm and data processing technology, as well as the advantages and challenges of these technologies in actual research. Despite challenges such as compatibility issues and data fusion complexities, advancements in fNIRs device optimization, standardized protocols, and computational methods are expected to address these obstacles. Future research should focus on refining this multimodal strategy to unlock its potential, enhancing the understanding of brain functions and improving diagnostic and therapeutic applications.
